# Institutional quality, aid flows, and malaria burden: a geospatial analysis of sub-Saharan Africa

**DOI:** 10.1186/s12936-025-05592-3

**Published:** 2025-10-14

**Authors:** Caroline Namubiru

**Affiliations:** Department of Economics, Faculty of Economics and Social Sciences, University of Tübingen, Tübingen, Germany

**Keywords:** Malaria, Spatial effects, Spatial panel data models

## Abstract

**Background:**

Malaria remains a major public health challenge in sub-Saharan Africa, accounting for approximately 95 percent of global malaria deaths despite extensive interventions. Significant disparities persist in disease burden across countries, with some achieving remarkable progress while others experiencing persistently high transmission rates, suggesting factors beyond resource availability influence disease control effectiveness. This study examines the relationship between institutional quality, development aid flows, and malaria burden across 38 sub-Saharan African countries during 2010–2022.

**Methods:**

The analysis employed malaria cases and deaths per 1,000 population as malaria burden measures. Key explanatory variables included development assistance for health per capita, government effectiveness indices and health worker density as institutional quality indicators, alongside control variables for intervention coverage, climatic factors, and socioeconomic conditions. Data was sourced from the World Health Organization, United Nations Development Program, Institute for Health Metrics and Evaluation, Malaria Atlas project, Demographic and Health surveys, and World Bank databases. Spatial econometric models, including Spatial Durbin Models, Spatial Lag of X, and Spatial Durbin Error Models, were used to account for spatial autocorrelation and cross-border transmission effects, while fixed-effects models with Driscoll-Kraay standard errors provided baseline estimates.

**Results:**

The study found overall malaria burden reduction across sub-Saharan Africa. Malaria cases and deaths demonstrated significant spatial autocorrelation annually, indicated by Moran's I statistics. Findings revealed that increased health worker density, enhanced institutional effectiveness, and higher aid levels are positively associated with the burden. These effects persisted with lagged values of health workers and government effectiveness. The geospatial analysis reveals that the malaria burden is driven more by local conditions with limited spillover effects from neighbouring countries.

**Conclusion:**

Findings highlight that while malaria burden has generally declined across sub-Saharan Africa, it is spatially clustered. There is need for localized health systems strengthening alongside targeted regional coordination.

**Supplementary Information:**

The online version contains supplementary material available at 10.1186/s12936-025-05592-3.

## Background

Malaria is one of the most significant public health burdens facing sub-Saharan Africa [1, 2] with the region accounting for 95 percent of global malaria deaths and 94 percent of the malaria cases despite having only 17 percent of the world's population [3]. Ending malaria is important for sustainable development. Sustainable development goal number three focuses on ensuring healthy lives and promotion of well-being for all at all ages with target 3.3 postulating that; By 2030, end the epidemics of AIDS, tuberculosis, malaria, and neglected tropical diseases and combat hepatitis, water-borne diseases, and other communicable diseases [4]. To achieve this target, coordinated efforts at global, regional, and national levels focus on controlling and eliminating malaria. Efforts include vector control and prevention, as well as medical interventions such as funding for diagnosis, prompt and effective treatment, intermittent preventive treatment in pregnancy, vaccines, and antimalarial drugs, complemented by public health policies and health-seeking behaviours [3, 5].

Significant progress has been made since 2000 with an estimated 2.2 billion cases of malaria and 12.7 million deaths averted [3]. Similarly, between 2000 and 2015, global malaria mortality declined by 60 percent [6], however, the rate of improvement has plateaued, raising questions about current control strategies' sustainability and effectiveness. Significant disparities exist in disease burden across SSA countries, with some achieving remarkable progress while others experience persistently high transmission rates. Countries like Rwanda have reduced malaria incidence by 88 percent since 2018 [7], others such as Nigeria and the Democratic Republic of Congo continue to carry over one-third of global malaria cases and deaths [3]. Institutional quality, funding, and the capacity to implement and coordinate health interventions play a critical role in determining health outcomes [8, 9]. Theoretically, North's work on institutional quality demonstrates that effective institutions reduce transaction costs, enhance predictability, and facilitate collective action which are all critical elements of successful disease control programmes [10]. Development assistance for health (DAH) constitutes a significant source of funding for malaria control in sub-Saharan Africa. Investment in malaria control reached over US$50 billion between 2000 and 2022. Global funding for malaria control and elimination totalled US$ 4.0 billion across 90 countries, slightly lower than the US$ 4.1 billion in 2022 but higher than the US$ 3.5 billion available in 2021 with the WHO African Region receiving 75 percent of this funding, owing to its high malaria burden [3]. Aid effectiveness is however influenced by institutional and governance factors, alignment issues between donor priorities and national health strategies [11–13] which determine absorptive capacity, transparency, and program implementation efficiency [14]. Health worker density is a critical component of health system capacity that directly affects malaria burden. A sufficient and well-distributed health workforce improves access to diagnosis, treatment, and prevention services, which are fundamental to reducing malaria morbidity and mortality [15]. However, disparities in health worker availability persist across many sub-Saharan African countries, exacerbating inequities in malaria outcomes [16].

Malaria is transmitted through the bites of female *Anopheles* mosquitoes [3], but the transmission process is shaped by environmental, socioeconomic, and geopolitical factors that vary across space and time. Drug and insecticide resistance, invasive vectors, conflicts, land use, climate change and disruptions to healthcare delivery spread in spatial patterns that cross national borders, requiring regional surveillance and coordinated responses [17–24]. Transmission risk is influenced by spatial spillover effects, where neighbouring conditions affect local outcomes [25, 26]. This study assesses how institutional quality, development assistance for health, and health system capacity, including health worker density, influence malaria burden in sub-Saharan Africa considering both temporal variations and spatial interdependencies between countries.

## Methods

### Data and sources

The study employed a quantitative, secondary-data longitudinal panel design covering the period 2010 to 2022 across 38 sub-Saharan African countries (see Fig. 2A, B for the list of included countries).

### Measurement of variables

#### Outcome variables

The outcome variables were Malaria cases and deaths scaled per 1000 population. Data was sourced from the 2024 World Malaria report [3].

#### Key explanatory variables

The key explanatory variables were:

Health worker density (per 100000 population) sourced from [3] as well as the Institute for Health Metrics and Evaluation [27]. The measure includes physicians, nurses, and midwives but excludes community health workers due to inconsistent reporting across countries.

Development Assistance for Health (DAH) in USD per capita from the [27] and cross-checked with the annual world malaria reports (World Health Organisation, 2012–2024). Funding values were deflated to 2022 constant US dollars using World Bank deflators and represent total health-related development assistance including both bilateral and multilateral flows.

Institutional quality was assessed using Political stability, corruption and government effectiveness indices from the World Governance Indicators (WGI) Database. Scores were rescaled from − 2.5 to + 2.5 to 0 to 1, with higher values indicating better outcomes. A composite institutional quality index was generated from the three indices using Principal component analysis (PCA). It is referred to in the text as government effectiveness. Data was confirmed suitable for factor analysis (overall Kaiser–Meyer–Olkin (KMO) = 0.6621; the KMO value for each variable exceeded the 0.5; Bartlett’s test of sphericity p = 0.000 < 0.05). The component, positively correlated with all three indices, explained 77.80 percent of the variance and had the highest eigenvalue.

#### Other variables

***Intervention variables:*** Bed net access (%), indoor residual spraying (IRS) coverage (%), Effective treatment for malaria (%) and antenatal care coverage (ANC4), defined as the proportion of women receiving four or more antenatal visits. Data were sourced from [27], the Malaria Atlas Project [28], and supplemented with Demographic and Health Survey (DHS) reports.

***Environmental factors***: Precipitation data in millimetres (mm) from the World Bank Climate Change Knowledge Portal [29].

***Economic and structural factors:*** Gross National Income (GNI) per capita from the United Nations Development Programme (UNDP), and urbanicity, defined as the proportion of the population residing in urban areas, from the IHME database [30].

In cases of missing data, additional comparisons were made using DHS and country-specific DHS reports [3, 28, 30] from various years. Interpolations using country-specific linear trends were applied where necessary. The variables, their sources and summary statistics are described in Table A1 in additional files.

### Conceptual model and hypotheses

The study adopts a modified health production function to analyse the effect of various determinants on malaria deaths and cases across countries. The model conceptualizes health outcomes as a function of both direct and indirect pathways, consistent with Grossman’s (1972) health production theory. The model is stipulated as:

$$H_{it} = f(M_{it} , I_{it} , A_{it} , G_{it} , E_{it} , S_{it} )$$

where ${H}_{it}$ represents health outcomes (malaria cases and deaths) for country i at time t; ${M}_{it}$ represents medical and non-medical interventions (health worker density, IRS coverage, ITN access, ANC4); ${I}_{it}$ represents institutional quality (government effectiveness); ${A}_{it}$ represents aid flows (DAH per capita); ${G}_{it}$ represents economic conditions (GNI per capita); ${E}_{it}$ represents environmental factors (precipitation); and ${S}_{it}$ represents structural factors (urbanisation). This model integrates both supply-side and contextual variables, recognizing that malaria outcomes result not only from health system capacity but also from broader socio-political and environmental environments.

### Data analysis

To analyse the data, (1) descriptive methods were applied, including summary statistics, line graphs, bar graphs, heat maps, and hotspot analysis. These visualized geographic clustering, temporal trends, and the distribution of malaria cases and deaths per 1,000 population across countries. For the regressions, both malaria outcomes (cases and deaths per 1,000) were log-transformed to address the scale effect and skewness in the data. (2) Baseline regression analysis that included pooled OLS to establish baseline relationships assuming homogeneity across countries and time periods; Panel fixed effects (FE) models to assess temporal variations in the relationships. The FE model was adopted as it was supported by the Hausman test (p < 0.05); and finally 1-year lag models [31], to capture delayed intervention effects. For this model, 1-year lags were applied to all explanatory variables except precipitation to capture delayed intervention effects. Diagnostic tests after the Panel FE model detected heteroskedasticity, cross-sectional dependence, and serial correlation, which were addressed mainly using Driscoll–Kraay standard errors with fixed effects were implemented. This technique produces standard errors robust to cross-sectional and temporal dependence [32]. Additionally, one-period lagged specifications were estimated to examine whether relationships differ when allowing for implementation delays. Further, panel regression models with Panel-Corrected Standard Errors and Robust Clustering to address panel-specific autocorrelation and heteroskedasticity. The standard errors were clustered at the country level to account for within-country correlation of observations over time while allowing for heteroskedasticity. Elasticities and marginal effects were calculated for key interventions (ITN access, IRS coverage, ANC4, health worker density, and DAH per capita) to quantify the percentage change in outcomes from a 1 percentage change in each variable.

(3) Spatial panel regression models were to assess the geospatial relationship of the variables with the malaria burden. These models were run after detecting spatial autocorrelation in the malaria outcomes using Global Moran's I statistics [33]. The Moran’s I statistic tests the null hypothesis that the malaria outcomes in one country are independent of those observed in neighbouring countries. The index can take values in the interval − 1 to 1, using a statistical significance test where significance is based on p-values.

Then the Local Moran’s I was used as a local indicator of spatial association (LISA). For each observed unit, the LISA provides information on the extent of significant spatial grouping of similar values around the observed unit. It detects statistically significant clusters and outliers [34]. Spatial dependence in malaria outcomes was identified (see Table A2 in the additional files) justifying estimating spatial panel models. Several spatial models including the Spatial Durbin Model (SDM), Spatial Durbin Error Model (SDEM) and, the spatial lag model of X (SLX) were used in the analysis after yielding lower AIC and BIC values compared to Spatial Autoregressive Model (SAR) and the spatial error model (SEM) though further analysis for robustness checks was based on the SDM as it has better coefficients for the Log-likelihood ratio test. The SDM incorporates both spatial lags of outcomes and explanatory variables, allowing for both spillover effects in outcomes and spatial spillovers in intervention effects. It tests whether interventions in neighbouring countries affect local outcomes, and it captures cross-border intervention spillovers. In the second phase, spatial panel models which support maximum likelihood for panel data were run [35]. The estimation of these models was done using a spatial weight matrix: the binary contiguity matrix (W) capturing neighbouring country relationships useful for modelling policy diffusion and regional interventions. This matrix was row-standardized and applied across the spatial panel models used in the analysis. The matrix is a square matrix representing the contiguity of countries. It lies between 0 and 1 and it is equal to one if any 2 countries have a mutual border, otherwise, it equals 0.

## Results

### Descriptive statistics

**Fig. 1:**
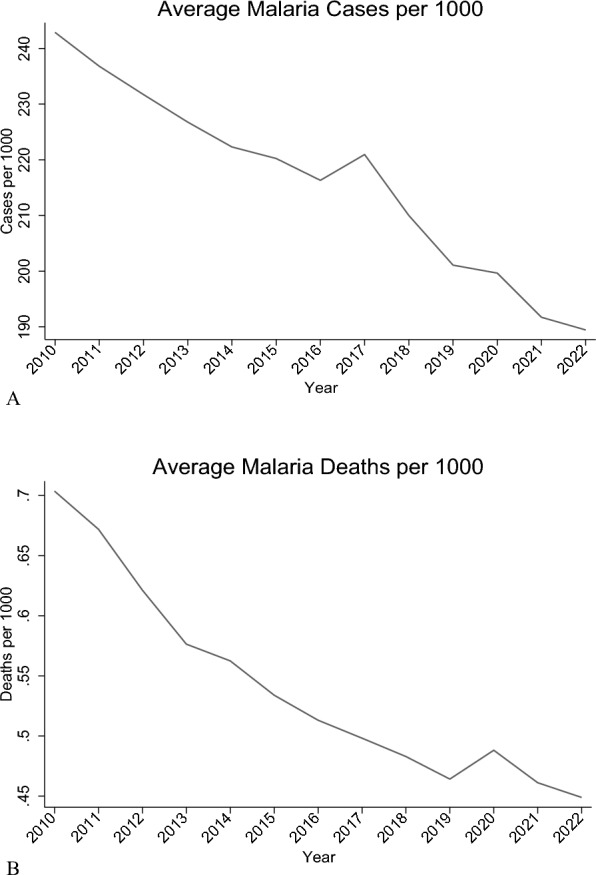
**A** Graph showing annual average malaria cases per 1000 (2010–2022). (Source: Author’s construction). **B** Graph showing annual average malaria deaths per 1000 (2010–2022). (Source: Author’s construction)

**Fig. 2:**
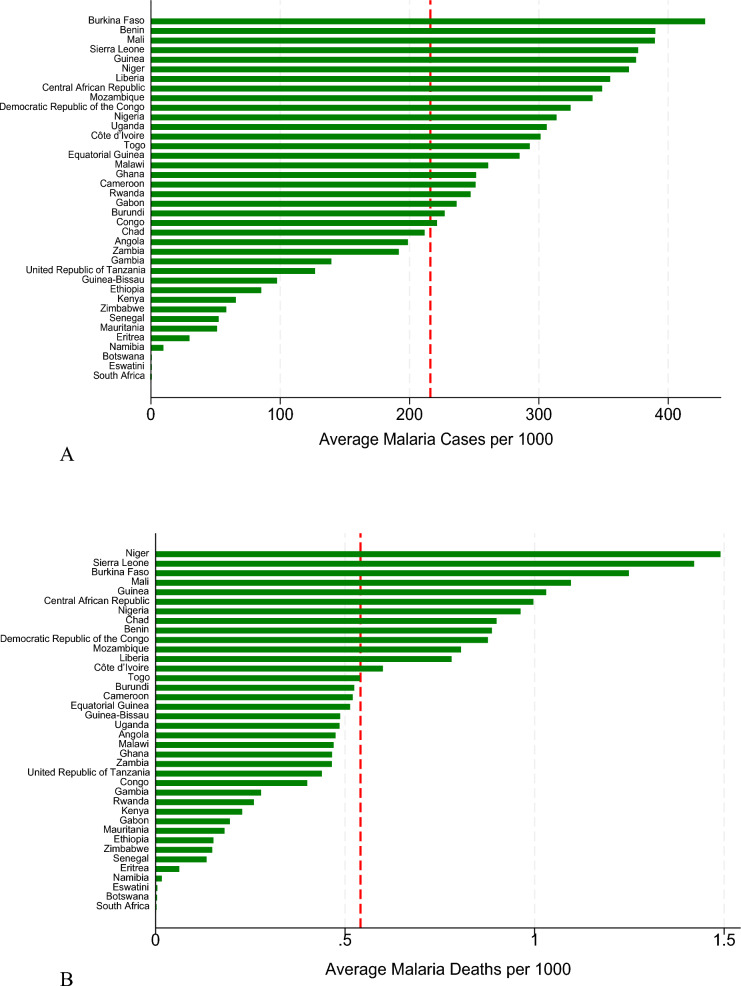
**A** Plot of mean malaria cases per 1000 (2010–2022). (Source: Author’s construction). **B** Plot of mean malaria deaths per 1000 (2010–2022). (Source: Author’s construction)

**Fig. 3:**
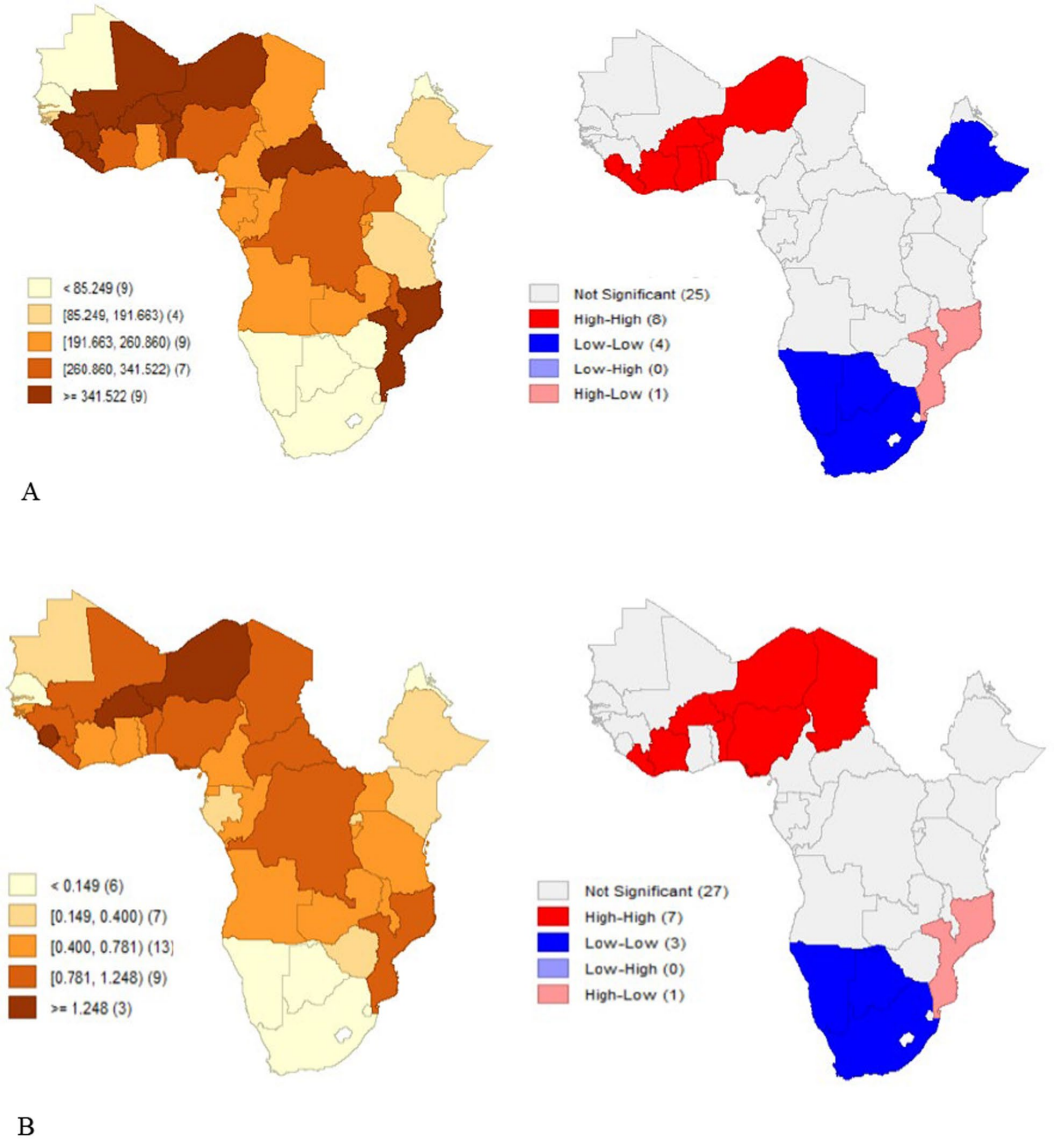
**A** Malaria cases per 1000. **B** Malaria deaths per 1000

Figure 1A, B show a consistent decline in annual average malaria cases and deaths per 1000 population from 2010 to 2022 in SSA. In Fig. 2A, B, countries in Western Africa (Burkina Faso, Niger, Sierra Leone) have significantly higher values for cases and deaths per 1000 while those in Southern Africa (Botswana, South Africa, Eswatini) have significantly low values. The hotspot analysis conducted to identify any existence of spatial clustering in the malaria outcomes is shown in Fig. 3A, B. There is a distinct spatial distribution of both malaria cases and malaria deaths. The western part of Africa has distinctively high cases and deaths averagely over time indicating a regional hotspot. This is indicated by the High-High clusters in red that show that countries with high malaria prevalence are surrounded by neighbours with similarly high values. The southern part has very low cases and deaths over time. High-Low outliers in pink in the south-eastern part of Africa indicates countries with high malaria burden surrounded by lower-burden neighbours, such as Mozambique which stands out with high cases and high deaths compared to the neighbours which may reflect localized health system or ecological differences. Similar results are reported by the hotspot map which shows concentration of high cases and deaths in the western part of Africa and low cases and deaths in the southern part of Africa.

### Baseline regression analysis of malaria cases and deaths

To assess the baseline regressions, panel regression models were estimated in addition to the pooled OLS and the lagged panel regression. The results (Fig. A1 in the additional files) show that the health worker density, DAH, GNI per capita, government effectiveness, ITN access and precipitation have a significant association with malaria cases and deaths. Indoor residual spraying (IRS) shows significant negative associations in the pooled OLS model. The lagged panel model yields similar results but finds no significant lagged effects for antenatal care coverage, IRS, or DAH.

To assess the relative magnitude of effects, elasticity estimates were calculated for key variables following the baseline regression estimates. Table 1 shows that a 1 percent increase in insecticide-treated net (ITN) access is associated with a 0.08 percent reduction in malaria cases and a 0.18 percent reduction in deaths, indicating a stronger protective effect against mortality. Health worker density shows negative elasticities for both outcomes, with a 1 percent increase associated with 0.56 percent and 0.93 percent reductions in cases and deaths respectively. However, the marginal effects for health worker density are positive, suggesting a simultaneous increase in detected cases. DAH per capita also shows negative elasticities (−0.29% for cases and −0.39% for deaths) but positive marginal effects. The discrepancy between negative elasticities and positive margins suggests that aid is often allocated in response to burden, with health benefits emerging over longer timeframes. Antenatal care coverage (ANC4) demonstrates strong and consistent protective elasticities, with a 1 percent increase linked to 1.94 percent reductions in both cases and deaths. Indoor residual spraying (IRS) coverage shows no significant effects in either elasticity or marginal estimates.

**Table 1:** Elasticies and Margins per percentage point change in intervention

Variables	Elasticity		Margins	
	Cases	Deaths	Cases	Deaths
ITN-access	−0.08* (0.04)	−0.18*** (0.04)	−0.01*** (0.01)	−0.01*** (0.01)
IRS coverage	−0.00 (0.01)	0.00 (0.01)	0.01 (0.01)	0.01 (0.01)
Health worker density per 100000	−0.56** (0.27)	−0.93*** (0.19)	7.31*** (1.22)	1.07 (0.79)
DAH per capita	−0.29** (0.11)	−0.39***(0.09)	0.53*** (0.06)	0.52*** (0.08)
ANC4	−1.94***(0.67)	−1.94*** (0.67)	−7.68*** (1.75)	−9.09*** (1.25)

### Panel regression analysis of malaria cases and deaths

Panel regression models using fixed effects with Driscoll–Kraay standard errors were used to address serial correlation, heteroskedasticity, and cross-sectional dependence. Additionally, panel regressions with panel-corrected standard errors and robust clustering were employed to ensure the robustness of the findings. The results in Tables 2 and 3 show that with regards to health worker density, there is a strong and significant positive association with malaria cases across all specifications except the panel correlated standard errors model. However, no significant association is found with malaria deaths. DAH, government effectiveness and precipitation show positive and significant association with both malaria cases and deaths. Antenatal care attendance and ITN access exhibits consistent negative associations with both malaria cases and deaths. Urbanization demonstrates strong protective effects against malaria cases. IRS coverage demonstrates no statistically significant association with either malaria incidence or mortality across all analytical specifications.

**Table 2:** Panel regression analysis for malaria cases per 1000

DV: malaria cases per 1000 (log)	(1)	(2)	(3)	(4)	(5)
	Driscoll-Kraay	Driscoll-Kraay with 1 lag	FE-Robust Country	Panel correlated SEs	FE-Robust
Health worker density (log)	6.98*** (1.34)	6.98*** (1.44)	6.98** (3.10)	4.54* (2.39)	6.98** (3.10)
IRS coverage	0.01 (0.00)	0.01 (0.01)	0.01 (0.00)	0.00 (0.01)	0.01 (0.00)
ITN-access	−0.00** (0.00)	−0.00** (0.00)	−0.00* (0.00)	0.01*** (0.00)	−0.00* (0.00)
DAH per capita (log)	0.54*** (0.06)	0.54*** (0.07)	0.54** (0.23)	0.55*** (0.11)	0.54** (0.23)
Precipitation (log)	0.45*** (0.14)	0.45** (0.15)	0.45** (0.21)	0.73*** (0.09)	0.45** (0.21)
Urbanicity	−49.04*** (10.57)	−49.04*** (11.86)	−49.04* (24.26)	−34.85* (20.83)	−49.04* (24.26)
ANC4	−7.83*** (1.74)	−7.83*** (2.01)	−7.83 (5.20)	−4.34 (3.49)	−7.83 (5.20)
GNIpc (log)	−0.63*** (0.11)	−0.63*** (0.11)	−0.63* (0.35)	−1.02*** (0.09)	−0.63* (0.35)
Government effectiveness index	0.37** (0.13)	0.37** (0.14)	0.37* (0.21)	−0.79*** (0.19)	0.37* (0.21)
Effective treatment	0.00 (0.01)	0.00 (0.01)	0.00 (0.01)	−0.01*** (0.00)	0.00 (0.01)
Observations	494	494	494	494	494
R-squared			0.12	0.63	0.12
Number of groups	38	38	38	38	38

Standard errors in parentheses

^***^p < 0.01, **p < 0.05, *p < 0.1

**Table 3:** Panel Regression Analysis for Malaria Deaths per 1000

DV: malaria deaths per 1000 (log)	(1)	(2)	(3)	(4)	(5)
	Driscoll-Kraay	Driscoll-Kraay with 1 lag	FE-Robust Country	Panel correlated SEs	FE-Robust
Health worker density (log)	1.37 (0.88)	1.37 (1.40)	1.37 (2.03)	−2.07 (2.77)	1.37 (2.03)
IRS coverage	0.00 (0.01)	0.00 (0.01)	0.00 (0.01)	−0.00 (0.00)	0.00 (0.01)
ITN-access	−0.00** (0.00)	−0.00** (0.00)	−0.00*** (0.00)	0.01*** (0.00)	−0.00*** (0.00)
DAH per capita (log)	0.52*** (0.08)	0.52*** (0.10)	0.52** (0.23)	0.50*** (0.13)	0.52** (0.23)
Precipitation (log)	0.30* (0.14)	0.30* (0.17)	0.30* (0.15)	0.50*** (0.09)	0.30* (0.15)
Urbanicity	−5.92 (7.14)	−5.92 (11.70)	−5.92 (18.10)	19.07 (24.62)	−5.92 (18.10)
ANC4	−8.96*** (1.36)	−8.96*** (1.72)	−8.96* (4.99)	−8.13* (4.17)	−8.96* (4.99)
GNIpc (log)	−0.50*** (0.15)	−0.50*** (0.15)	−0.50** (0.24)	−0.94*** (0.11)	−0.50** (0.24)
Government effectiveness index	0.12 (0.07)	0.12 (0.07)	0.12 (0.17)	−0.64*** (0.14)	0.12 (0.17)
Effective treatment	−0.00 (0.01)	−0.00 (0.01)	−0.00 (0.01)	−0.01*** (0.00)	-−0.00 (0.01)
Observations	494	494	494	494	494
R-squared			0.20	0.38	0.20
Number of groups	38	38	38	38	38

Standard errors in parentheses

^***^p < 0.01, **p < 0.05, *p < 0.1

### Spatial regression analysis of malaria cases and deaths

Before utilizing spatial regression models, spatial autocorrelation in malaria cases and deaths was assessed using Moran’s I statistics. In Table A2 in the additional file, the Z scores and p-values clearly state that the spatial autocorrelation effects in malaria outcomes per 1000 are significant at a 5% level across all the years. The positive Moran’s *I* values indicates that the countries with high malaria cases and deaths per 1000 (countries in the high-high groups) tend to locate together, like the low burden countries (countries in the low-low groups). For malaria deaths per 1000, there exists persistent and moderately strong positive spatial autocorrelation across SSA countries for the study period. The values consistently range between 0.36 and 0.46, suggesting that countries with high malaria mortality are geographically clustered together, and so are countries with low incidence. From 2010 to 2014, Moran’s I values remain relatively stable around 0.36, indicating moderate but consistent clustering of malaria outcomes during these years, but increase above 0.40 after 2014. Regarding malaria cases per 1000, there exists moderate positive spatial autocorrelation from 2010 to 2022. The results of the LISA cluster analysis using Local Moran’s I are shown in Fig. 3A, B. Significant positive values indicate hotspots (high-high clusters), while significant negative values indicate cold spots (low-low clusters).

The local effects from the spatial regression models align with results obtained from the Driscoll-Kraay FE models. Table 4 shows that higher health worker density, DAH and precipitation are associated with increased malaria cases and deaths. Higher values of government effectiveness are associated with higher values of malaria cases. ANC, GNIpc and ITN access exhibit negative associations with malaria cases and deaths. IRS Coverage and effective treatment show no significant association with malaria outcomes. Further, Health worker density and DAH in neighbouring areas have negative and significant effects on local malaria deaths, suggesting cross-border benefits of health workforce availability and funding. In contrast, antenatal care coverage in neighbouring areas shows a positive and significant association (about 20) with local malaria deaths and cases, possibly reflecting spatial clustering of health service access or underlying risk factors. For malaria cases, spatial spillovers are weaker. Among the lagged variables, only antenatal care coverage remains positively significant. Malaria cases respond more strongly to local conditions, with weak spatial spillovers compared to mortality. The Wald tests of spatial terms are significant (p < 0.05), confirming presence of spatial effects. The spatial lag coefficients of the dependent variables and spatial error terms are not statistically significant, indicating that the malaria outcomes in one country are not directly dependent on the malaria outcomes in neighbouring countries once covariates and their spatial lags are accounted for and that the explanatory variables and their spatial lags capture most of the spatial variation in the data.

**Table 4:** Spatial regression models

Variables	DV: Malaria deaths per 1000 (log)			DV: Malaria cases per 1000 (log)		
	SLX	SDM	SDEM	SLX	SDM	SDEM
Health worker density (log)	3.90* (2.31)	3.96* (2.32)	3.94* (2.37)	8.59*** (2.73)	8.45*** (2.73)	8.93*** (2.93)
IRS coverage	0.00 (0.00)	0.00 (0.00)	0.00 (0.00)	0.01* (0.00)	0.01* (0.00)	0.01* (0.00)
ITN-access	−0.00** (0.00)	−0.00** (0.00)	−0.00** (0.00)	−0.01** (0.00)	−0.00** (0.00)	−0.00** (0.00)
DAH per capita (log)	1.13*** (0.34)	1.13*** (0.34)	1.12*** (0.34)	1.01** (0.40)	1.01** (0.40)	1.01** (0.40)
Precipitation (log)	0.33** (0.15)	0.32** (0.15)	0.33** (0.15)	0.44*** (0.17)	0.44*** (0.17)	0.46*** (0.18)
Urbanicity	−8.61 (20.11)	−9.18 (20.12)	−9.13 (20.07)	−55.76** (24.21)	−53.55** (23.70)	−57.52** (25.62)
ANC4	−23.79*** (5.77)	−23.72*** (5.77)	−23.67*** (5.81)	−17.88*** (6.87)	−18.55*** (6.81)	−18.16*** (6.95)
GNIpc (log)	−0.48*** (0.14)	−0.48*** (0.14)	−0.48*** (0.14)	−0.66*** (0.17)	−0.64*** (0.17)	−0.65*** (0.17)
Government Effectiveness Index	0.13 (0.11)	0.14 (0.11)	0.13 (0.11)	0.36*** (0.13)	0.37*** (0.13)	0.36*** (0.13)
Effective treatment	0.01 (0.01)	0.01 (0.01)	−0.01 (0.01)	0.01 (0.01)	0.01 (0.01)	0.01 (0.01)
W*Health Worker Density (log)	−3.02*** (1.03)	−3.00*** (1.03)	−3.01*** (1.04)	−1.09 (1.22)	−1.34 (1.23)	−1.29 (1.26)
W*ITN-Access	−0.00 (0.00)	−0.00 (0.00)	−0.00 (0.00)	−0.00 (0.00)	−0.00 (0.00)	−0.00 (0.00)
W*DAH per capita (log)	−0.79** (0.40)	−0.82** (0.40)	−0.79** (0.40)	−0.62 (0.47)	−0.66 (0.47)	−0.61 (0.48)
W*ANC4	20.29*** (6.12)	20.49*** (6.12)	20.19*** (6.16)	14.37** (7.24)	15.87** (7.31)	14.99** (7.37)
W* Government Effectiveness	−0.16 (0.27)	−0.15 (0.27)	−0.16 (0.27)	−0.22 (0.32)	−0.21 (0.32)	−0.22 (0.33)
W*Malaria deaths per 1000 people		0.05 (0.09)			0.12 (0.09)	
W*Malaria cases per 1000 people						
e.log_malaria_deaths_per_1000			0.04 (0.09)			
e.log_malaria_cases_per_1000						0.11 (0.09)
Wald Chi2	127.84***	129***	121.9***	78.15***	80.25***	72.60***
Wald test of spatial terms Chi2	12.44**	12.78**	12.49*	12.96**	14.71**	13.90**
Log-likelihood	−77.55	−77.39	−77.46	−153.04	−152.32	−152.23
AIC	187.10	188.78	188.91	338.09	338.46	338.64
BIC	254.35	260.23	260.35	405.33	409.91	410.08

Standard errors in parentheses, 38 countries, n = 494

^***^p < 0.01, **p < 0.05, *p < 0.1

The different robustness checks run confirm the validity of the main results. First, a model was run incorporating potential structural breaks that could have been caused by COVID-19 by excluding data from 2020 and 2021. The unprecedented global disruption caused by the COVID-19 pandemic significantly affected health systems and disease surveillance infrastructure. Then, the exclusion of Southern African countries was tested to see whether results hold across different regional epidemiological contexts, given their lower malaria endemicity and distinct health system characteristics. Because real-world relationships between health system variables and disease outcomes are rarely strictly linear. Quadratic terms for health worker density and DAH were included to assess any effect due to diminishing returns and potential non-linear relationships. Health worker density, DAH, and governance effectiveness effects on malaria burden are robust across model variations and time periods. However, the results are not robust to the case fatality ratio (CFR), the ratio of malaria deaths to reported cases as an alternative outcome to examine health system performance. The results are shown in table S3 and table S4 in the supplementary materials.

## Discussion

DAH shows a positive association with malaria burden, indicating targeted aid allocation to high-burden countries and improved reporting due to donor-supported monitoring systems [14, 36–39]. Additional funding of aid produces greater health improvements in countries [40]. However, the findings contrast with previous studies where targeted DAH is associated with reductions in malaria mortality [41, 42], the effectiveness of such assistance is likely contingent upon national governance quality, institutional readiness, and the timeliness of implementation. Government effectiveness correlates positively with malaria case detection, reinforcing the view that improved governance enhances surveillance and reporting systems rather than directly reducing transmission or mortality [43]. Health worker density increasing the malaria burden reflects the role of health systems in both detecting and treating malaria, where improved surveillance leads to higher observed incidence despite overall reductions in disease severity and mortality [16, 44]. This pattern aligns with prior studies showing that stronger health systems can inflate disease burden metrics through better detection [5]. Insecticide-treated nets (ITNs) access and antenatal care coverage (ANC4) have a negative relationship with malaria cases and deaths in sub-Saharan Africa [45]. ITN access has a role in preventing malaria transmission and severe outcomes [46–48]. Indoor residual spraying (IRS) coverage showing no significant relationship with malaria outcomes likely reflects operational challenges, subnational heterogeneity in implementation, and growing insecticide resistance, which undermine IRS effectiveness [49, 50]. Environmental factors confirm established epidemiological patterns, with precipitation increasing malaria burden through creating favourable conditions for vector breeding and transmission cycles [51–53]. Urbanization lowers malaria incidence by improving housing and reducing vector habitats, but its inconsistent effects on mortality suggest disparities in healthcare access or treatment quality within urban populations [24, 54, 55]. GNI per capita demonstrates consistent negative relationships with both malaria incidence and mortality, confirming the fundamental role of economic development in disease burden reduction. Higher income levels facilitate improvements in housing quality, nutritional status, healthcare access, and public health infrastructure that collectively reduce malaria risk and improve treatment outcomes [56, 57]. Higher health worker density and development assistance in surrounding regions reduce local malaria mortality, indicating cross-border health benefits. The positive association of antenatal care coverage in neighbours with local malaria deaths and cases likely reflects spatial clustering of risk factors or reporting patterns. Spatial spillovers in malaria cases are less pronounced, suggesting that cases respond more to local conditions. The potential limitations of the study include the study’s restriction to national-level panel data for the set of health system and intervention variables over the study period. It does not assess intervention quality, community-level factors, or entomological indicators. Future research should integrate subnational panel data to capture geographic heterogeneity. The study examines only aggregate development assistance without distinguishing between intervention types, limiting the ability to identify which funding streams are most effective. Future research should disaggregate aid data to assess the relative impacts.

## Conclusion

The study concludes that malaria cases and deaths are primarily influenced by local factors, with limited direct spatial dependence between neighbouring countries’ malaria outcomes. Elasticity results show that ITN access, health worker density, antenatal care coverage, and development assistance for health are associated with reductions in malaria cases and deaths, though higher health worker density, development assistance for health, government effectiveness and precipitation increase local malaria burden, while antenatal care coverage, income levels, and insecticide-treated net access reduce it basing on the marginal effects. Spatial spillovers from neighbouring countries show that increased health worker density and funding nearby reduce local malaria deaths, indicating regional benefits of resource availability. On the contrary, higher antenatal care coverage in neighbouring countries is associated with increased local malaria burden. Therefore, spatial models confirm the existence of spatial effects with malaria outcomes responding more to local conditions than to neighbouring countries’ malaria outcomes. Therefore, malaria control strategies should focus on strengthening local health systems and regional resource sharing, while recognizing spatial patterns in health service coverage and environmental risk.

## Supplementary Information

Supplementary material 1.

Supplementary material 2.

## Data Availability

All data supporting the findings of this study are publicly available. Malaria burden data and different indicators available in the public domain at World Health Organization (https://www.who.int/data), Malaria Atlas Project https://data.malariaatlas.org, Institute for Health Metrics and Evaluation (https://www.healthdata.org), DHS data (http://dhsprogram.com/data/available-datasets.cfm), and climate data available at https://climateknowledgeportal.worldbank.org/. Development Assistance for Health data available at Institute for Health Metrics and Evaluation (https://ghdx.healthdata.org/ihme_data). Institutional quality data available in the public domain at World Governance Indicators Database (https://info.worldbank.org/governance/wgi/).
