# Supplementary material for: Institutional quality, aid flows, and malaria burden: a geospatial analysis of sub-Saharan Africa
Source: Malar J. 2025 Oct 14;24:332. doi: 10.1186/s12936-025-05592-3 (PMC12519830; doi:10.1186/s12936-025-05592-3)
Supplement: Supplementary file 1 — Supplementary material 1. [file 12936_2025_5592_MOESM1_ESM.docx]

**Supplementary materials**

**Table S1: Baseline regression results (Panel FE and Pooled OLS regressions)**

|  | **Malaria Cases per 1000 (log)** | | **Malaria Deaths per 1000 (log)** | |
| --- | --- | --- | --- | --- |
| **Variables** | **FE** | **Pooled OLS** | **FE** | **Pooled OLS** |
|  |  |  |  |  |
| Health Worker Density (log) | 6.98*** | -5.78 | 1.37 | -10.31 |
|  | (2.58) | (9.07) | (2.18) | (7.28) |
| IRS Coverage | 0.01* | -0.01 | 0.00 | -0.02** |
|  | (0.00) | (0.01) | (0.00) | (0.01) |
| ITN-Access | -0.00** | 0.04*** | -0.00** | 0.03*** |
|  | (0.00) | (0.00) | (0.00) | (0.00) |
| DAH per capita (log) | 0.54*** | 0.54 | 0.52*** | 0.49 |
|  | (0.19) | (0.70) | (0.16) | (0.56) |
| Precipitation (log) | 0.45** | 0.65*** | 0.30** | 0.38*** |
|  | (0.18) | (0.09) | (0.15) | (0.08) |
| Urbanicity | -49.04** | 30.61 | -5.92 | 67.60 |
|  | (23.27) | (84.22) | (19.71) | (67.60) |
| ANC4 | -7.83* | -2.62 | -8.96** | -5.06 |
|  | (4.21) | (15.76) | (3.57) | (12.65) |
| GNIpc (log) | -0.63*** | -0.50*** | -0.50*** | -0.49*** |
|  | (0.17) | (0.10) | (0.14) | (0.08) |
| Government Effectiveness Index | 0.37*** | -0.84*** | 0.12 | -0.80*** |
|  | (0.14) | (0.15) | (0.12) | (0.12) |
| Effective treatment | 0.00 | -0.02*** | -0.00 | -0.02*** |
|  | (0.01) | (0.01) | (0.01) | (0.00) |
|  |  |  |  |  |
| Observations | 494 | 494 | 494 | 494 |
| R-squared | 0.12 | 0.59 | 0.20 | 0.63 |
| Number of countries | 38 | 38 | 38 | 38 |

Standard errors in parentheses

*** p<0.01, ** p<0.05, * p<0.1

**Table S2: Panel Regression for Malaria Cases and Deaths per 1000 based on lagged values**

|  | **(1)** | **(2)** |
| --- | --- | --- |
| **Variables** | **Malaria cases** | **Malaria Deaths** |
|  |  |  |
| L.Health Worker Density (log) | 8.62*** | 4.90** |
|  | (2.54) | (2.20) |
| L.IRS Coverage | 0.00 | -0.00 |
|  | (0.00) | (0.00) |
| L.ITN-Access | -0.00* | -0.00** |
|  | (0.00) | (0.00) |
| L.DAH per capita (log) | -0.09 | -0.14 |
|  | (0.19) | (0.17) |
| Precipitation (log) | 0.28 | 0.28* |
|  | (0.18) | (0.16) |
| L.Urbanicity | -73.00*** | -45.02** |
|  | (22.92) | (19.88) |
| L.ANC4 | 3.12 | 4.10 |
|  | (4.38) | (3.80) |
| L.GNIpc (log) | -0.66*** | -0.50*** |
|  | (0.18) | (0.16) |
| L.Government Effectiveness Index | 0.54*** | 0.22* |
|  | (0.15) | (0.13) |
| Effective treatment | 0.01 | -0.01 |
|  | (0.01) | (0.01) |
|  |  |  |
| Observations | 456 | 456 |
| R-squared | 0.11 | 0.14 |
| Number of ctry | 38 | 38 |

Standard errors in parentheses

*** p<0.01, ** p<0.05, * p<0.1

**Table S3: Robustness tests, Malaria cases per 1000**

|  |  | **DV: Malaria cases per 1000 (log)** | | |
| --- | --- | --- | --- | --- |
|  | **DV: Case Fatality Ratio** | **SDM** | **Exclusion of COVID-time** | **No Southern Africa** |
|  |  |  |  |  |
| Health Worker Density (log) | -0.03 | 8.43*** | 8.96*** | 4.57*** |
|  | (0.02) | (2.75) | (2.92) | (0.64) |
| Health Worker Density (log)^2 |  | -0.01* |  |  |
|  |  | (0.00) |  |  |
| DAH per capita (log) ^2 |  | 0.00 |  |  |
|  |  | (0.00) |  |  |
| IRS Coverage | 0.00 | 0.01* | 0.01** | 0.01 |
|  | (0.00) | (0.00) | (0.00) | (0.00) |
| ITN-Access | 0.00 | -0.00*** | -0.00** | -0.00 |
|  | (0.00) | (0.00) | (0.00) | (0.00) |
| DAH per capita (log) | 0.01 | 0.36 | 1.08** | 0.21*** |
|  | (0.01) | (1.04) | (0.43) | (0.05) |
| Precipitation (log) | -0.00 | 0.48*** | 0.38* | 0.19 |
|  | (0.00) | (0.17) | (0.20) | (0.14) |
| Urbanicity | 0.42** | 142.35 | -55.08** | -33.67*** |
|  | (0.18) | (105.67) | (25.36) | (4.96) |
| ANC4 | -0.14*** | -22.99*** | -18.94*** | -3.10** |
|  | (0.05) | (8.23) | (7.19) | (1.39) |
| GNIpc (log) | 0.00 | -0.67*** | -0.63*** | -0.60*** |
|  | (0.00) | (0.17) | (0.20) | (0.08) |
| Government Effectiveness Index | -0.00 | 0.37*** | 0.46*** | 0.30** |
|  | (0.00) | (0.13) | (0.16) | (0.13) |
| Effective treatment | -0.00 | 0.01 | 0.01 | 0.01 |
|  | (0.00) | (0.01) | (0.01) | (0.01) |
| W*Health Worker Density (log) | -0.02** | -1.05 | -1.37 |  |
|  | (0.01) | (1.22) | (1.31) |  |
| W*Government effectiveness | -0.00 | -0.00 | -0.00 |  |
|  | (0.00) | (0.01) | (0.01) |  |
| W*ITN-Access | 0.00 | -0.00 | -0.00 |  |
|  | (0.00) | (0.00) | (0.00) |  |
| W*DAH per capita (log) | -0.01 | -0.69 | -0.77 |  |
|  | (0.01) | (0.47) | (0.51) |  |
| W*ANC4 | 0.11** | 15.11** | 15.96** |  |
|  | (0.05) | (7.30) | (7.71) |  |
| W*DV | -0.10 | 0.11 | 0.13 |  |
|  | (0.11) | (0.09) | (0.10) |  |
|  |  |  |  |  |
| Observations | 494 | 494 | 418 | 442 |
| Number of groups | 38 | 38 | 38 | 34 |
| Wald Chi2 | 14.09 | 84.13 | 61.13 |  |
| Prob > Chi2 | 0.339 | 0.000 | 0.000 |  |

Standard errors in parentheses

*** p<0.01, ** p<0.05, * p<0.1

**Table S4: Robustness tests, Malaria deaths per 1000**

|  | **(2)** | **(3)** | **(4)** |
| --- | --- | --- | --- |
| **DV: Malaria deaths per 1000 (log)** | **SDM** | **Exclusion of COVID-time** | **No Southern Africa** |
|  |  |  |  |
| Health Worker Density (log) | 3.92* | 3.85 | 0.75 |
|  | (2.31) | (2.46) | (0.99) |
| Health Worker Density (log)^2 | -0.01** |  |  |
|  | (0.00) |  |  |
| DAH per capita (log) ^2 | 0.00 |  |  |
|  | (0.00) |  |  |
| IRS Coverage | 0.00 | 0.00 | 0.00*** |
|  | (0.00) | (0.00) | (0.00) |
| ITN-Access | -0.00*** | -0.00** | -0.00** |
|  | (0.00) | (0.00) | (0.00) |
| DAH per capita (log) | 0.42 | 1.21*** | 0.16*** |
|  | (0.88) | (0.36) | (0.04) |
| Precipitation (log) | 0.35** | 0.24 | 0.26*** |
|  | (0.15) | (0.17) | (0.08) |
| Urbanicity | -28.48*** | -8.36 | -8.10 |
|  | (6.96) | (21.35) | (7.48) |
| ANC4 | -28.48*** | -23.18*** | -1.49 |
|  | (6.96) | (6.05) | (1.31) |
| GNIpc (log) | -0.50*** | -0.48*** | -0.56*** |
|  | (0.14) | (0.17) | (0.12) |
| Government Effectiveness Index | 0.14 | 0.13 | 0.06 |
|  | (0.11) | (0.13) | (0.04) |
| Effective treatment | -0.01 | -0.01 | -0.01 |
|  | (0.01) | (0.01) | (0.01) |
| W*Health Worker Density (log) | -2.74*** | -2.94*** |  |
|  | (1.04) | (1.10) |  |
| W*Government effectiveness | -0.13 | -0.19 |  |
|  | (0.27) | (0.34) |  |
| W*ITN-Access | -0.00 | -0.00 |  |
|  | (0.00) | (0.00) |  |
| W*DAH per capita (log) | -0.82** | -0.91** |  |
|  | (0.40) | (0.43) |  |
| W*ANC4 | 19.10*** | 20.02*** |  |
|  | (6.10) | (6.41) |  |
| W*Malaria cases per 1000 people | 0.03 | 0.05 |  |
|  | (0.09) | (0.10) |  |
|  |  |  |  |
| Observations | 494 | 418 | 442 |
| Number of groups | 38 | 38 | 34 |
| Wald Chi2 | 134.7 | 97.84 |  |
| Prob > Chi2 | 0.000 | 0.000 |  |

Standard errors in parentheses

*** p<0.01, ** p<0.05, * p<0.1
