# Supplementary material for: Institutional quality, aid flows, and malaria burden: a geospatial analysis of sub-Saharan Africa
Source: Malar J. 2025 Oct 14;24:332. doi: 10.1186/s12936-025-05592-3 (PMC12519830; doi:10.1186/s12936-025-05592-3)
Supplement: Supplementary file 2 — Supplementary material 2. [file 12936_2025_5592_MOESM2_ESM.docx]

**Table A1: Variables, Measurements, Definitions, and Descriptive Statistics.**

| **Variables** | **Unit of measurement** | **Definition of the variables** | **Obs** | **mean** | **sd** | **min** | **p50** | **max** |
| --- | --- | --- | --- | --- | --- | --- | --- | --- |
| Malaria deaths per 1000 | Rate | Malaria deaths per 1000 population | 494 | 0.54 | 0.43 | 0.001 | 0.46 | 2.37 |
| Malaria cases per 1000 | Rate | Malaria cases per 1000 population | 494 | 216.14 | 139.97 | 0.03 | 231.61 | 709.79 |
| Government effectiveness | Index rescaled from − 2.5 to + 2.5 to range from 0 to 1 | The index of government effectiveness captures political stability, rule of law and government effectiveness. Source: World Bank Governance Indicators | 494 | 0.44 | 0.22 | 0 | 0.44 | 1 |
| Health Worker Density | Ratio | Number of employed health workers (of any specialty) per 100,000 population | 494 | 41.59 | 5.03 | 34.15 | 41.34 | 49.92 |
| DAH percapita | Amount | DAH per person (2022 USD) | 494 | 19.92 | 4.01 | 16.5 | 18.18 | 29.76 |
| Precipitation | Precipitation | Annual precipitation in mm. Source: WB Climate Change Knowledge Portal | 494 | 94.28 | 50.14 | 7.76 | 88.58 | 240.85 |
| LGNIpc |  | Gross National Income percapita | 494 | 7.95 | 0.79 | 6.57 | 7.69 | 9.84 |
| ITN-access | Percentage | Percentage of population accessing ITNs/LLIns | 494 | 43.77 | 23.79 | 0 | 46.71 | 91.25 |
| ANC4 | Proportion | Proportion of pregnant women receiving 4 or more antenatal care visits including 1 or more from a skilled provider | 494 | 0.59 | 0.02 | 0.56 | 0.58 | 0.65 |
| Effective Treatment | Percentage | Effective treatment in 100 malaria cases | 494 | 43.07 | 12.82 | 12.20 | 43.04 | 71.66 |
| IRS coverage | Percentage | Percentage of households covered with Indoor Residual Spraying | 494 | 4.21 | 6.96 | 0 | 0.99 | 42.14 |
| Urbanicity | Proportion | Proportion of Urban Population | 494 | 0.26 | 0.02 | 0.24 | 0.26 | 0.29 |
| Years of Education | Count | Average Years of Education | 494 | 5.04 | 2.38 | 1 | 5 | 12 |

**Table A2: Malaria Cases and Deaths per 1000 Global Moran’s I statistics.**

| **Malaria Deaths per 1000** | | | |  |  |  |  |  |  |  |  |  |  |
| --- | --- | --- | --- | --- | --- | --- | --- | --- | --- | --- | --- | --- | --- |
| **year** | **2010** | **2011** | **2012** | **2013** | **2014** | **2015** | **2016** | **2017** | **2018** | **2019** | **2020** | **2021** | **2022** |
| **Moran’s I** | 0.3607 | 0.3863 | 0.3605 | 0.3596 | 0.3609 | 0.4103 | 0.4378 | 0.4576 | 0.4603 | 0.458 | 0.4282 | 0.4209 | 0.4134 |
| **Z-Score** | 3.251 | 3.4579 | 3.2256 | 3.2165 | 3.2035 | 3.6312 | 3.8662 | 3.9964 | 4.0198 | 3.9993 | 3.7617 | 3.6931 | 3.6383 |
| **p-value** | 0.0012 | 0.0005 | 0.0013 | 0.0013 | 0.0014 | 0.0003 | 0.0001 | 0.0001 | 0.0001 | 0.0001 | 0.0002 | 0.0002 | 0.0003 |
| **Malaria Cases per 1000** | | | |  |  |  |  |  |  |  |  |  |  |
| **Moran’s I** | 0.3855 | 0.3705 | 0.3355 | 0.3162 | 0.3472 | 0.3466 | 0.3733 | 0.3073 | 0.3861 | 0.4136 | 0.3908 | 0.3725 | 0.3462 |
| **Z-Score** | 3.3673 | 3.2431 | 2.9579 | 2.8029 | 3.0565 | 3.0523 | 3.2652 | 2.8137 | 3.3723 | 3.589 | 3.4052 | 3.2544 | 3.0413 |
| **p-value** | 0.0008 | 0.0012 | 0.0031 | 0.0051 | 0.0022 | 0.0023 | 0.0011 | 0.0049 | 0.0007 | 0.0003 | 0.0007 | 0.0011 | 0.0024 |

**Figure A1: Baseline Regression results**


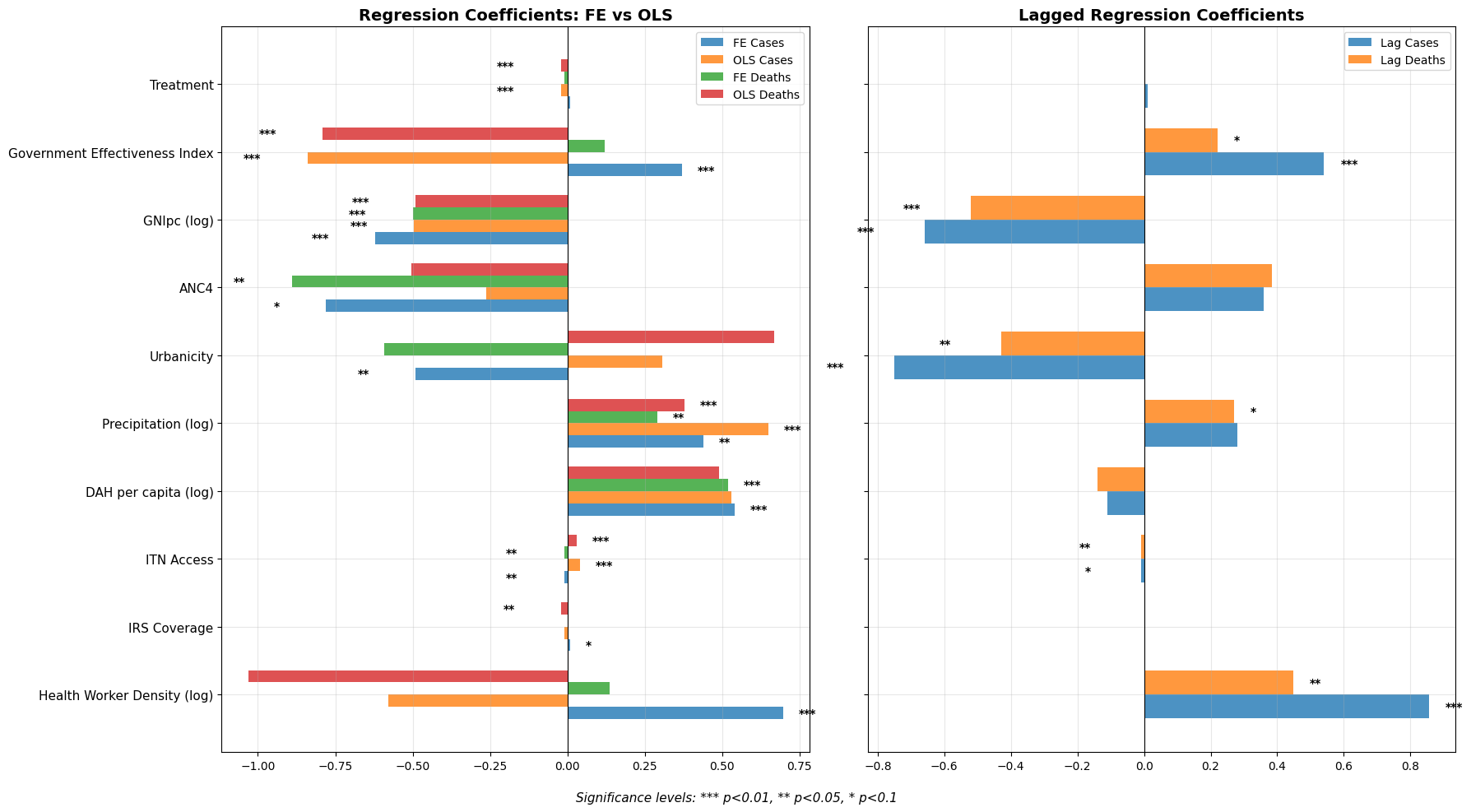


Note: the coefficients for health worker density and ANC4 are expressed/10 and the one for urbanicity is expressed as/100
